# The physical characteristics of human proteins in different biological functions

**DOI:** 10.1371/journal.pone.0176234

**Published:** 2017-05-01

**Authors:** Tengjiao Wang, Hailin Tang

**Affiliations:** 1 Department of Bioinformatics, Second Military Medical University, Shanghai, P.R. China; 2 Department of Biological Biodefense (Microbiology), Faculty of Tropical Medicine and Public Health, Second Military Medical University, Shanghai Key Laboratory of Medical Biodefense, Shanghai, P.R.China; Tulane University Health Sciences Center, UNITED STATES

## Abstract

The physical properties of gene products are the foundation of their biological functions. In this study, we systematically explored relationships between physical properties and biological functions. The physical properties including origin time, evolution pressure, mRNA and protein stability, molecular weight, hydrophobicity, acidity/alkaline, amino acid compositions, and chromosome location. The biological functions are defined from 4 aspects: biological process, molecular function, cellular component and cell/tissue/organ expression. We found that the proteins associated with basic material and energy metabolism process originated earlier, while the proteins associated with immune, neurological system process *etc*. originated later. Tissues may have a strong influence on evolution pressure. The proteins associated with energy metabolism are double-stable. Immune and peripheral cell proteins tend to be mRNA stable/protein unstable. There are very few function items with double-unstable of mRNA and protein. The proteins involved in the cell adhesion tend to consist of large proteins with high proportion of small amino acids. The proteins of organic acid transport, neurological system process and amine transport have significantly high hydrophobicity. Interestingly, the proteins involved in olfactory receptor activity tend to have high frequency of aromatic, sulfuric and hydroxyl amino acids.

## Introduction

The physical properties of proteins are the foundation of their biological function, and correspondingly the biological functions have selection constrains on the physical properties. Currently, there have been some work to explore the intrinsic relationships between physical properties and biological functions of proteins, for example, between isoelectric point and subcellular localization [1–3], between protein stability and biological processes [4–6] *etc*. But these separated studies could not provide us a comprehensive global view. In other hand, with the rapid development of functional genomics and systems biology, large amount of data have been accumulated, such as sequence [7], advance structure, post-translation modification [8], chromosome location, subcellular localization, biological process, tissue expression [9], associated diseases [10, 11], mRNA and protein abundance [12, 13], interacting proteins [14–16] *etc*. It becomes necessary and possible to make a comprehensive analysis of the relationships between these physical properties and biological functions.

In this study, we focused on 16 primary physical properties including origin time, evolutionary pressure, mRNA stability, protein stability, molecular weight (MW), hydrophobicity, isoelectric point (*p*I), 8 amino acid (AA) categories (non-polar, polar without charges, negative charged, positive charged, small, aromatic, sulfuric and hydroxyl) and chromosome location. The biological function of proteins were defined from 4 aspects: 3 are from Gene Ontology (GO) [17] that biological process (BP), molecular function (MF), and cellular component (CC), and the fourth is the cell/tissue/organ expression (CTOE). Concretely, 202 BP, 90 MF, 62 CC and 68 CTOE items were selected. Considering the high relevance between BP and MF, we mainly concerned BP, and treated MF as a supplement. We not only explored the 16 primary physical properties separately, but also some of their combinations, that are ⑴ origin time and evolutionary pressure; ⑵ mRNA and protein stability; ⑶ amino acids and protein MW.

## Materials and methods

### Simple sequence properties

Amino acids were categorized as follows: non-polar (A, V, L, I, P, F, W and M); polar without charge (G, S, T, C, Y, N and Q); negative charged (D and E); positive charged (H, K and R); small (A, C, D, G, N, P, S, T and V); aromatic (F, H, W and Y); sulfuric (C and M) and hydroxyl (S, T and Y). The proportion of amino acid group of protein is calculated as the residue number of a certain group divided by the total residue number of protein. Molecular weight (MW) of protein was calculated as the sum of molecular weight of each amino residue and additional 18 (H_2_O). Hydrophobicity for proteins was calculated as the sum of hydrophobicity values by using the Kyte and Doolittle index [18], and divided by the number of residue of protein sequence. Compete *p*I/Mw of ExPASy was used to calculate protein *p*I (**S1 File.**).

The molecular weight, hydrophobicity/hydrophilic and acidity/alkalinity characteristics of human proteins in different function categories were determined by their median values (see **S2 File**) and their distributions of each functions category with total proteins **(Figure A-C in S10 File**). There were 20 301, 4 005, 1 891 and 2 278 paired comparisons for BP, MF, CC and CTOE respectively. We quantified the statistical significance of each comparison by calculating the Bonferroni correction *P*-values (rank sum test) which (*P*-values < 0.05) were shown in **S3, S4, S5**and **S6 Files**. Additionally, the Bonferroni correction *p*-values for the comparison between each BP, MF, CC and CTOE class and total were shown in **S2**File, The isoelectric point characteristics of human proteins in different function categories were determined by analyzing their enrichments in each Isoelectric point class (< 7 group, >7 group, < 6 group and >9 group). The enrichment was measured by the ratio and significant level (Fisher exact test, **S7 File**). The distribution and median of the proportion of non-polar, negative charged, positive charged, small, aromatic, sulfuric (C and M) and hydroxyl (S, T and Y) amino acid were calculated in each function item (**Figure D-K in S10 File**). The amino acid compositions (small, sulfur-containing, aromatic etc) of proteins are mainly determined by the structural class, especially the membrane structure. Thus, we added the overlap proteins comparison of the intrinsic to membrane (CC), intrinsic to plasma membrane (CC) with the classes with the higher amino acid composition, hydrophobicity, pI, and plotted the distribution curves of each class (S11 File). We compared the overlap proteins among the classes with most highest small, aromatic, sulfur, hydroxy amino acid composition, and the *p*-values (rank sum test) between total and the classes (excluding the common protein). The results were shown in S12 File.

### mRNA and protein decay rates

Yang *et al*. estimated the mRNA decay rate in HepG2 cell line with 5,245 GeneBank Accessions assignment [19]. We transformed these 5,245 GeneBank Accessions into 4,953 Entrez Gene IDs through DAVID [20] and then transformed these Entrez Gene IDs into 4,464 Uniprot IDs. Yen *et al*. estimated proteins stabilities index (PSI) of 6,528 genes in 293T cell line [4]. We use the reciprocal of PSI to represent protein decay rate, and got 6,373 proteins assignment after id transition from Entrenz Gene ID to Uniprot Accession.

### Protein origin time and evolution pressure

The origin time of proteins are evaluated through comparing the homolog genes in the phylostratigraphic. If a human protein has a homologue in specie which fuses with *homo sapien* in evolution tree at time *T*, the origin time of the ancestor protein must be earlier than *T*. Species with orthologous can be used as fusion thread, and bifurcation points of evolution tree can be used as time marks. We first constructed a evolution tree from ref., [21]. Five origin time classes are distinguished. Then, we calculated the origin times of each protein based on their orthologous existence in other species and obtained 6,776 proteins with estimated origin time. The orthologous information was obtained from OrthoMCL [22].

The origin time characteristics of human proteins in different function categories were determined by analyzing their enrichments in each origin time class. The enrichment was measured by the ratio and significant level (Fisher exact test, **S8 File**). The FDR was estimated by comparing the *p*-values of 1,000 times random testing.

Evolution pressure which were represented by *K*a/*K*s [23]. The *K*a/*K*s values were from ref. [24]. The human-mouse protein *K*a and *K*s were downloaded from H-InvDB. The human-mouse orthologues comes from Inparanoid. Finally, we obtained 12,023 proteins with *K*a/*K*s.

### GO terms and cells/tissues/organs expression

The GO dataset was downloaded from http://www.geneontology.org/GO.downloads.shtml, dated June 2010. GOA (gene_association.goa_human, 2010.9.27) provided the GO annotation of proteins. Here, we referred to GO:0008150 (biological_process), GO:0003674 (molecular_function) and GO:0005575 (cellular_component) as level 0. We selected the BP terms in level 3–5, and discarded the terms that contain “negative regulation” or “positive regulation” words. In addition, we manually deleted 10 redundant BP terms (GO:0044429, GO:0044433, GO:0044445, GO:0005911, GO:0050794, GO:0060341, GO:0000075, GO:0051239, GO:0007187 and GO:0010033). For CC and MF terms, the selected terms demanded following three conditions: 1) Belongs to level 3 or higher level; 2) Contained no less 100 proteins; 3) The number of contained proteins was less than half of its parent terms. Eventually, we obtained 202 BP, 90 MF and 62 CC items.

Tissue expression information in Swissprot is annotated as TISSUE in the comment lines RC. There were total 470 tissue items. We selected 68 items that contain no less than 100 proteins to insure the statistics power.

### Chromosome groups

The 24 human chromosomes can also be further divided to 48 short arms and 121 cytobands (with more than 100 protein coding genes). The chromosome location information of proteins were obtained from the NCBI. The chromosome location characteristics of human proteins in different function categories were determined by analyzing their enrichments in each chromosome location class (S9 File). 35 BP, 17 CC and 17 CTOE items were enriched in at least one chromosome (*p*<0.001), about 15.84%,27.42%, 25.00% of total items respectively.

## Results

### Origin time, evolution pressure

Most proteins associated with basic genetic, and energy metabolism process have early origin time (>4 billion years, Gya): translation (93.33%), nucleobase, nucleoside, nucleotide and nucleic acid transport (93.10%), microtubule-based movement (88.46%), and electron transport chain (88.00%). While most proteins associated with immune, chemotaxis and neurological system process have late origin time (<0.45, and 0.45–0.99Gya). Four CC items, including ribosome (91.53%), spliceosomal complex (90.91%), mitochondrial matrix (90.32%) and mitochondrial membrane part (82.85%) have more than 80% proteins in the earliest class (>4Gya). Three items, MHC protein complex (100%), intermediate filament (64.10%), and external side of plasma membrane (57.89%) have the highest proportion of latest originated proteins (<0.45Gya). Interestingly, most proteins from neurological system process originated 0.45Gya, except Cajal-Retzius cell 96.55% of proteins originated before 4Gya. The proteins associated with peripheral blood and immune tissue originate later. The proteins within 4 carcinomas that ovarian, cervix, colon and mammary carcinomas have relative early origin time.

The evolution pressure characteristics of human proteins in different function categories were determined by comparing the *K*a/*K*s median value of each functions category **(S2 File)**. We also compared the *K*a/*K*s distribution of proteins between total and each functions category (**Figure L in S10 File**). The proteins involving in immune, defense, and chemotaxis tend to have high evolution pressure, suggesting that the main aspect for human evolution at present is to fight against pathogens. The proteins associated with cytokine binding and hormone activity have high evolution pressure. The extracellular cytokines experience higher evolution pressure than intracellular ones, suggesting that extracellular cytokines play more important role to response the extracellular stimulus. Thus, we should pay more attention to the extracellular part when exploring the approaches of preventing some disease, such as pathogen invading, cancer, and among others. Tissues related digestion such as small intestine and stomach have high evolution pressure. It maybe results from the diverse human diet. The rapid evolution of trachea and tongue may be influenced by the emergence of human language.

**Fig 1:**
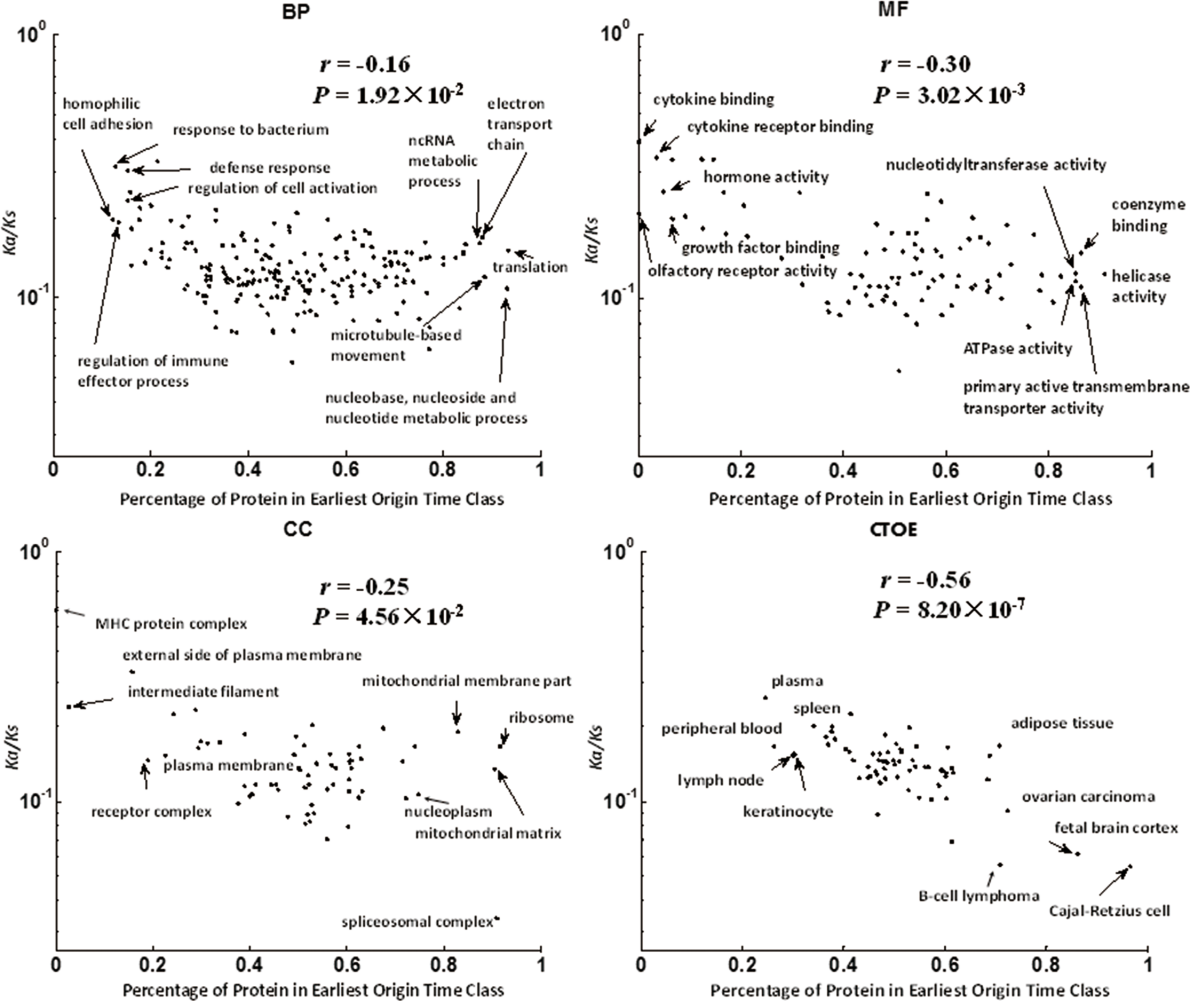
Scatter plot of median of *K*a/*K*s versus percent of proteins in earliest origin time (>4Gya) class for BP, MF, CC and CTOE. Data points represent the different function items. The function items with high proportion of old proteins tend to have lower *K*a/*K*s, especially in CTOE. Spearman Rank Test was used to examine the correlation between *K*a/*K*s and the percent of proteins in earliest class. The correlation coefficient *r* and *P*-value were shown. The names of items with top and bottom 5% of proteins in earliest origin time classes were shown.

Several studies have shown that the young proteins are under higher evolution pressure than old proteins [25, 26]. It is also confirmed in our work (**Figure M in S10 File**). The function items with high proportion of young proteins tend to have high evolution pressure (**Fig 1**), especially high negative correlation in CTOE (*r* = -0.57, *P* = 8.20×10^−7^). It may suggest that tissue level is the scale of biosystem that influences the evolution pressure strongly.

### mRNA, protein stability

**Fig 2:**
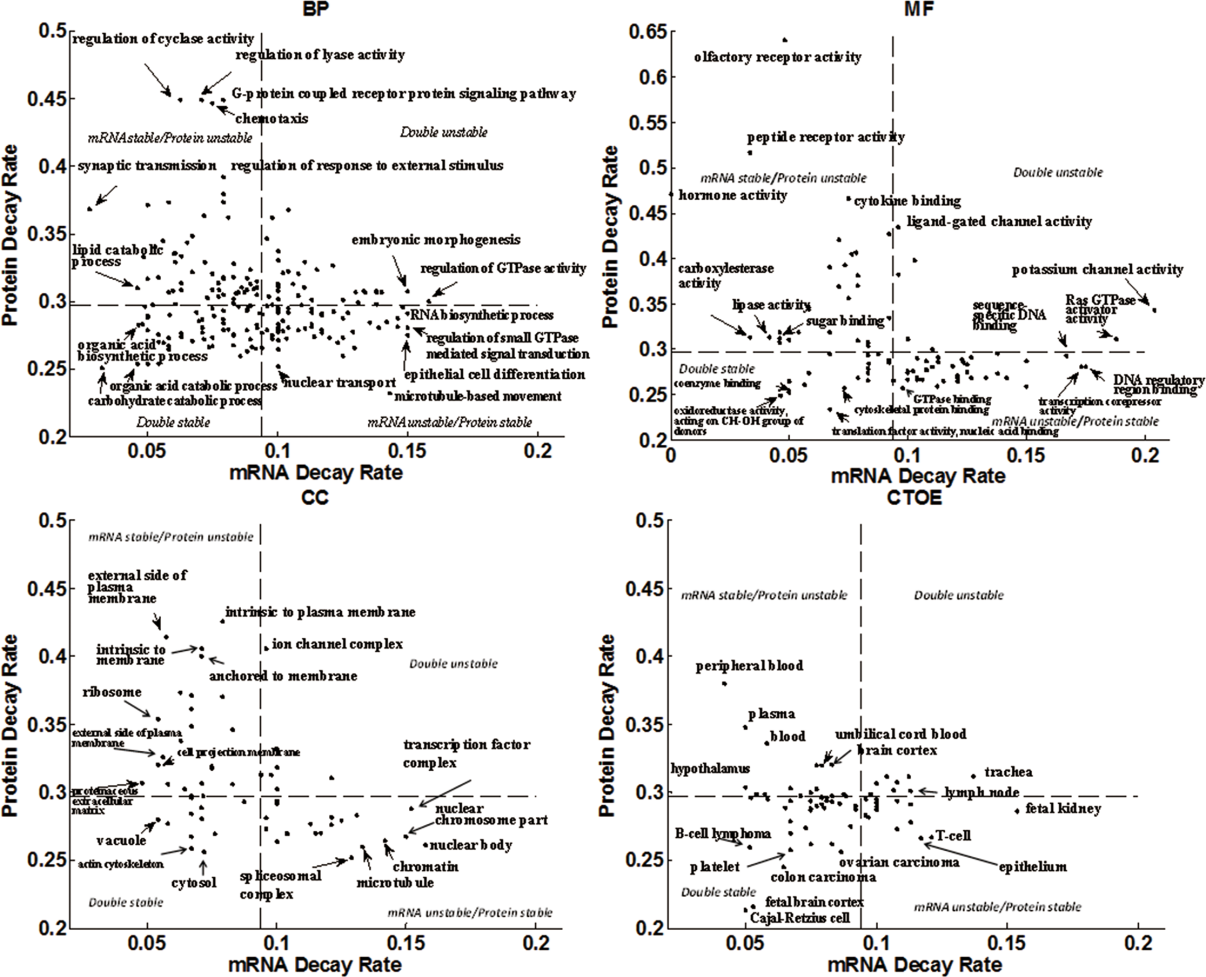
Scatter plot of median of mRNA decay rate versus median of protein decay rate of function items. Data points represent the different function items. The stability plane was divided into 4 zones: double-stable, mRNA unstable/protein stable, mRNA stable/protein unstable, and double-unstable. There were few function items falling into the double-unstable zone.

Combinations of mRNA and protein stability were formed under functional constraints in the process of evolution. We made a scatter plot of median of mRNA decay rate versus median of protein decay rate of each BP, MF, CC and CTOE items respectively and divided the stability plane into 4 zones (**Fig 2**). The median of mRNA and protein decay rates (0.09 and 0.30) are set as boundary value respectively.

Energy and basic materials metabolism (including carbohydrate catabolic, lipid catabolic and cellular amino acid derivative metabolic), B-cell lymphoma, fetal brain cortex and Cajal-Retzius cell tend to be double-stable. Double stabilities of mRNA and protein can improve the efficiency of metabolic enzyme usage, and meet the heavy demand of metabolic enzymes. Synaptic transmission, immune, peripheral cell proteins (e.g., MHC complex, outside of plasma membrane and receptor complex), and blood related (e.g., peripheral blood, plasma and blood) are mRNA stable/protein unstable. High protein renew rate can ensure the sensitive response of synaptic transmission and rapid renewal of blood circulation. Structural proteins in nucleus (including nuclear body, spliceosomal complex, microtubule, nuclear chromosome, chromatin, and among others) are mRNA unstable/protein stable. There are few function items falling into the double-unstable zone, suggesting that the gene products, which are unstable both in mRNA and protein level, are not dominated in biosystem.

There exists a high negative correlation between mRNA and protein stability in CC (*r* = -0.50, *P* = 2.90×10^−5^) and weak correlation in MF (*r* = -0.35, *P* = 8.11×10^−4^), but not in BP (*r* = -0.11, *P* = 0.10) and CTOE (*r* = -0.01, *P* = 0.96). It may indicate that subcellular level is the right scale of biosystem that mRNA and protein stability co-regulate intensively.

### Molecular weight, small amino acids composition

**Fig 3:**
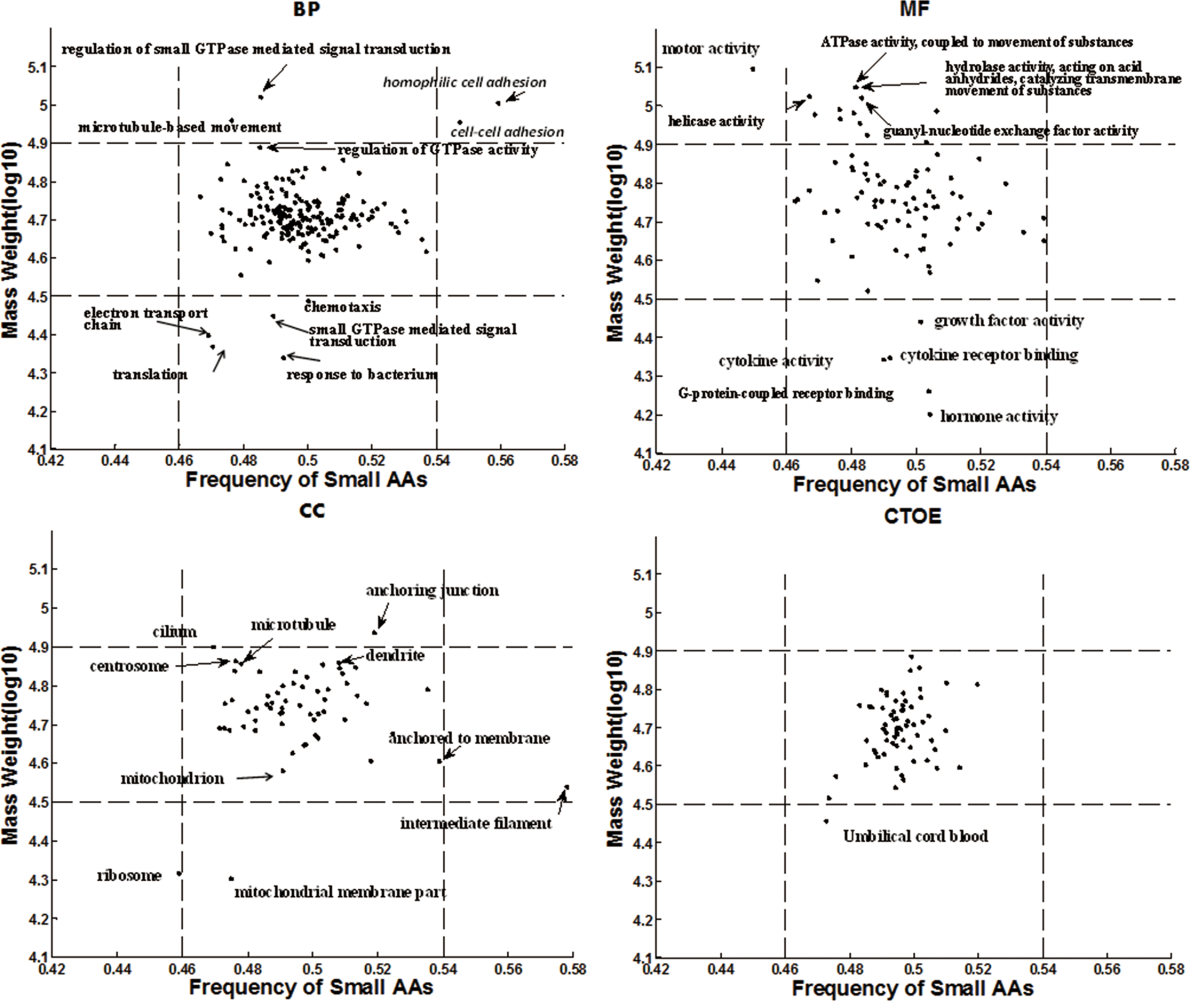
Scatter plot of median values of frequencies of small AA versus median values of MW of function items. The proteins from cell adhesion have high MW and high frequency of small AAs.

The proteins associated with regulation processes (*e*.*g*., response to bacterium, electron transport chain, small GTPase mediated signal transduction, chemotaxis, neurological system process, generation of precursor metabolites and energy, defense response, locomotory behavior, regulation of cell activation) are relatively small. The proteins associated with structure, movement (*e*.*g*., cell adhesion, cell morphogenesis, microtubule-based movement, cell part morphogenesis, microtubule cytoskeleton organization, membrane invagination, anatomical structure homeostasis, protein localization) tend to be larger. Compartmental functional organelles (*e*.*g*., mitochondrion, endosome and ribosome *etc*.) tend to be consisted of small proteins. Dispersive structural subcellular components (*e*.*g*., cilium, centrosome, dendrite, microtubule, neuron projection *etc*.) tend to be consisted of large proteins. Cell adhesion process (including homophilic cell adhesion and cell-cell adhesion) consists of large proteins with high proportion of small amino acid compositions (Fig 3, Table A in S11 File).

### Hydrophobicity, isoelectric point, polar, charged, sulfuric, hydroxyl and aromatic amino acids

The plasma membrane-related cellular components (*e*.*g*., intrinsic to membrane, intrinsic to plasma membrane, basolateral plasma membrane, apical plasma membrane *etc*.) tend to have high hydrophobicity, while nuclear proteins including spliceosomal complex, nuclear body, chromosome, chromatin and nucleolus are opposite. We found that three BP items (organic acid transport, amine transport, and neurological system process) and three MF items (metal ion transmembrane transporter activity, peptide receptor activity, and olfactory receptor activity) have significant high hydrophobicity distribution (**Figure C in S10 File**). All 6 classes have higher overlap with intrinsic to membrane (Table E in S11 File, Figure R in S11 File), but there were significant difference compared to the intrinsic to membrane (*P*-values <10^−10^). 98% proteins of olfactory receptor activity (MF) also are part of neurological system process (BP).

The median *p*I of most items are less than 7. The proteins in the ribosome, intrinsic membrane, and mitochondrion tend to be alkaline, especially the ribosome which contains up to 79.59% proteins with *p*I>9. Ribosome has lower overlap proteins with intrinsic membrane (Table H in S12 File). Previous study have shown that membrane proteins were more basic [2], but we found that some membrane-related cellular components (*e*.*g*., membrane raft and membrane-bounded vesicle) also are acidic. The “homophilic cell adhesion” has the highest proportion (96.40%, *p* = 0) of acidic proteins among BP items, and the next two are “microtubule-based movement” and “Golgi vesicle transport”, which have more than 75% acidic proteins. There are lower overlap proteins among these three classes (Table I in S12 File). There were nine CTOE items with more than half alkaline proteins. Four CTOE items have more than 70% of acidic proteins, that are fetal brain cortex (81.64%), Cajal-Retzius cell (79.06%), plasma (73. 20%) and platelet (71.64%) (**S7 File**).

Proteins associated with receptor activity have high proportion of hydroxyl amino acids. Especially, the “olfactory receptor activity” item has higher proportion of aromatic, sulfuric and hydroxyl amino acids than any other MF items (**Figure C-D in S10 File**).

### Chromosome location

The “homophilic cell adhesion” (44.60%, *p* = 0) and “cell-cell adhesion” (22.60%, *p* = 0) were enriched in the proteins in chromosome 5. Next was the “neurological system process” in chromosome 11 (22.60%, *p* = 0).

The MHC protein complex proteins were enriched in chromosome 6 (93.22%, *p* = 0). Furthermore, MHC protein complex proteins were main in the short arm of chromosome 6 (6p2), up to 88.13%. About 77.17% intermediate filament proteins located in chromosome 12, 17 and 21.

The “Blood”, “Peripheral blood” and “B-Cell” were enriched in chromosome 6. It was worth noting that about 19.63% proteins of “Neuroblastoma” located in chromosome 14.

## Discussion

We examined the physical property distributions of diverse functional groups. The proteins expectedly involved in primary genetic, material and energy metabolic processes originated earlier, while the proteins involved in immune and neurological system process originated later. Interestingly, we found most proteins from Cajal-Retzius cell have the earliest origin time.

Genes may have evolved specific combinations of mRNA and protein lives under functional constraints [27–29]. mRNA and protein stability had been studied separately due to the technical limitations. Recently, with the mature of protein decay rate measuring technology [30], researchers had pay more attention to the combination of mRNA and protein stability. Schwanhausser *et al*. had measured absolute mRNA and protein abundance and turnover by parallel metabolic pulse labeling for more than 5,000 genes in mammalian cells [31]. They showed that the proteins associated with translation, respiration and central metabolism are mRNA and protein double-stable. The proteins participating in the processing of mRNAs, tRNAs and non-coding RNAs are mRNA unstable/protein stable. Extracellular proteins are mRNA stable/protein unstable. Transcription factors, signaling genes, chromatin modifying enzymes and genes with cell-cycle-specific functions are mRNA and protein double-unstable. In our study, there are few double-unstable items, very different with Schwanhausser’s results. We thought that more mRNA and protein double-unstable data from same sample are needed to support these results. Furthermore, mRNA and protein expression level should be considered so that a more detailed dynamic model depicting the relationship between mRNA and protein, expression level and stability will achieved. The genome-wide studies of the stability of mRNA and protein are only in its infancy. A great progression will be made in next few years [28, 29].

MW, hydrophobicity and *p*I are the basic properties of proteins. We showed that proteins associated with cell adhesion process are consisted of large protein with high proportion of small amino acids. Ribosome proteins have the highest alkalinity and strong hydrophobicity, resulting from its high proportion of hydrophoibcity AAs and positive charged AAs, and low proportion of negative charged AAs.

## Supporting information

S1 File Protein property data. Values of 20283 proteins for 14 kinds of properties and classification of proteins.
(XLS)

S2 File Sorted function groups by median values. Different function categories were sorted through by the median values of molecular weight, hydrophobicity/hydrophilic and, acidity/alkalinity and amino acid components respectively.
(XLS)

S3 File Less than 0.5 Bonferroni correction *P*-Values for function categories (BP). We quantified the statistical significance of each comparison among BP categories by calculating the Bonferroni correction *P*-values.
(XLSX)

S4 File Less than 0.5 Bonferroni correction *P*-Values for function categories (CC). (XLSX)

S5 File Less than 0.5 Bonferroni correction *P*-Values for function categories (MF). (XLSX)

S6 File Less than 0.5 Bonferroni correction *P*-Values for function categories (CTOE). (XLSX)

S7 File *p*I enrichment analysis. The isoelectric point characteristics of different function categories were determined by analyzing their enrichments in each Isoelectric point class.
(XLS)

S8 File Origin time enrichment analysis. (XLS)

S9 File Chromosome enrichment analysis. (XLS)

S10 File **Fig. A-M.** Properties values distribution of function items with top, bottom 10 median mass weight value and total for CC, BP, MF and CTO.
(DOCX)

S11 File **Table A-F, Fig N-S.** Protein overlaps between the intrinsic membrane proteins and the classes with higher hydrophobicity, *p*I, amino acid composition.
(DOCX)

S12 File **Table G-M.** *P*-values of rank sum test between total and other classes with the higher hydrophobicity, *p*I, amino acid composition.
(DOCX)
