# Supplementary material for: The physical characteristics of human proteins in different biological functions
Source: PLoS One. 2017 May 1;12(5):e0176234. doi: 10.1371/journal.pone.0176234 (PMC5411090; doi:10.1371/journal.pone.0176234)
Supplement: S10 File — Fig. A-M. Properties values distribution of function items with top, bottom 10 median mass weight value and total for CC, BP, MF and CTO. (DOCX) [file pone.0176234.s010.docx]

**Supp Fig. A-M**

**Fig. A** The mass weight distribution of function items with top(red), bottom(green) 10 median mass weight value and total for CC, BP, MF and CTO. The probability density curves were obtained by Gaussian-kernel smoothing of the individual data points.

**Fig. B** The *p*I distribution of function items with top(red) , bottom(green) 10 median *p*I value and total for CC, BP, MF and CTO. The probability density curves were obtained by Gaussian-kernel smoothing of the individual data points.

**Fig. C** The hydrophobicity distribution of function items with top(red) , bottom(green) 10 median hydrophobicity value and total for CC, BP, MF and CTO. The probability density curves were obtained by Gaussian-kernel smoothing of the individual data points.

**Fig. D** The distribution of frequency of polar amino acids with negative charges for function items with top(red) , bottom(green) 10 median value and total for CC, BP, MF and CTO. The probability density curves were obtained by Gaussian-kernel smoothing of the individual data points.

**Fig. E** The distribution of frequency of polar amino acids with positive charges for function items with top(red), bottom(green) 10 median value and total for CC, BP, MF and CTO. The probability density curves were obtained by Gaussian-kernel smoothing of the individual data points.

**Fig. F** The distribution of frequency of polar amino acids without charges for function items with top(red), bottom(green) 10 median value and total for CC, BP, MF and CTO. The probability density curves were obtained by Gaussian-kernel smoothing of the individual data points.

**Fig. G** The distribution of frequency of nonpolar amino acids for groups with top(red), bottom(green) 10 median Ka/Ks value and total for CC, BP, MF and CTO. The probability density curves were obtained by Gaussian-kernel smoothing of the individual data points.

**Fig. H** The distribution of frequency of small amino acids with negative charges for function items with top(red), bottom(green) 10 median value and total for CC, BP, MF and CTO. The probability density curves were obtained by Gaussian-kernel smoothing of the individual data points.

**Fig. I** The distribution of frequency of aromatic amino acids for function items with top(red), bottom(green) 10 median value and total for CC, BP, MF and CTO. The probability density curves were obtained by Gaussiankernel smoothing of the individual data points.

**Fig. J** The distribution of frequency of sulfuric amino acids for function items with top(red), bottom(green) 10 median value and total for CC, BP, MF and CTO. The probability density curves were obtained by Gaussiankernel smoothing of the individual data points.

**Fig. K** The distribution of frequency of hydroxyl amino acids for function items with top(red), bottom(green) 10 median value and total for CC, BP, MF and CTO. The probability density curves were obtained by Gaussian kernel smoothing of the individual data points.

**Fig. L** The Ka/Ks(log10) distribution of groups with top(red), bottom(green) 10 median value and total for CC, BP, MF and CTO. If the number of proteins with Ka/Ks values of a function item were less 40, this item could not be showed because of the statistics significance. The probability density curves were obtained by Gaussiankernel smoothing of the individual data points.

**Fig. M** The distribution of proteins Ka/Ks values for five origin time classes. The distributions are fitted by norm distribution. The earliest origin time class has the least average Ka/Ks value and the latest origin time class has the largest average Ka/Ks value.
