# Supplementary material for: The physical characteristics of human proteins in different biological functions
Source: PLoS One. 2017 May 1;12(5):e0176234. doi: 10.1371/journal.pone.0176234 (PMC5411090; doi:10.1371/journal.pone.0176234)
Supplement: S11 File — Table A-F, Fig N-S. Protein overlaps between the intrinsic membrane proteins and the classes with higher hydrophobicity, pI, amino acid composition. (DOCX) [file pone.0176234.s011.docx]

**Table A Protein overlaps between the intrinsic membrane proteins and the classes with higher small amino acid composition**

|  | intrinsic membrane (CC) (5385) | intrinsic to plasma membrane (CC) (1040) |
| --- | --- | --- |
| intrinsic to plasma membrane (CC)  (1040) | 1040  (8.53×10^-26^) |  |
| anchored to membrane (CC)  (132) | 132  (1.25×10^-26^) | 42  (2.25×10^-10^) |
| intermediate filament (BP)  (184) | 3  (1.85×10^-26^) | 2  (2.07×10^-16^) |
| cell-cell adhesion (BP)  (292) | 222  (7.60×10^-49^) | 66  (6.88×10^-21^) |
| homophilic cell adhesion (BP) (139) | 138  (3.18×10^-45^) | 35  (1.11×10^-25^) |

The values in the brackets are the P values of rank sum test between the classes in the first column and the intrinsic membrane, intrinsic to plasma membrane classes (excluding the common proteins). The numbers at the end of each class are the protein numbers of corresponding class.

**Fig. N The small amino acid composition distribution of the total (blue), intrinsic membrane, intrinsic plasma membranes (purple) and other classes (dark red). The probability density curves were obtained by Gaussian-kernel smoothing of the individual data points.**

**Table B Protein overlaps between the intrinsic membrane proteins and the classes with higher aromatic amino acid composition**

|  | intrinsic to membrane (CC) (5,385) | intrinsic to plasma membrane (CC)(1,040) |
| --- | --- | --- |
| lipid biosynthetic process (BP) (329) | 179  (6.55×10^-11^) | 17  (9.62×10^-13^) |
| neurological system process (BP) (947） | 657  (2.88×10^-29^) | 96  (1.45×10^-21^) |
| glycosylation  (BP) (114) | 100  (3.40×10^-11^) | 6  (8.66×10^-15^) |
| UDP-glycosyltransferase activity (MF) (126) | 113  (9.78×10^-11^) | 3  (5.60×10^-15^) |
| peptide receptor activity(MF)( 114) | 113  (4.42×10^-08^) | 70  (1.00×10^-12^) |
| transferase activity, transferring glycosyl groups (MF) (261) | 192  (1.52×10^-18^) | 8  (9.68×10^-23^) |
| olfactory receptor activity (MF) (422) | 422  (2.40×10^-104^) | 6  (3.99×10^-96^) |

The values in the brackets are the P values of rank sum test between the classes in the first column and the intrinsic membrane, intrinsic to plasma membrane classes (excluding the common proteins). The numbers at the end of each class are the protein numbers of corresponding class.

**Fig. O The aromatic amino acid composition distribution of the total (blue), intrinsic membrane, intrinsic plasma membranes (purple) and other classes (dark red). The probability density curves were obtained by Gaussian-kernel smoothing of the individual data points.**

**Table C Protein overlaps between the intrinsic membrane proteins and the classes with higher sulfur amino acid composition**

|  | intrinsic to membrane (CC)(5385) | intrinsic to plasma membrane (CC)(1,040) |
| --- | --- | --- |
| response to bacterium (BP) (134) | 27  (3.56×10^-8^) | 20  (1.23×10^-6^) |
| chemotaxis (BP) (173) | 64  (1.00×10^-8^) | 33  (3.20×10^-7^) |
| neurological system process (BP) (147) | 44  (8.99×10^-5^) | 16  (7.27×10^-6^) |
| G-protein-coupled receptor binding (MF) (141) | 29  (2.79×10^-6^) | 14  (2.48×10^-5^) |
| receptor activity (MF) (1,715) | 1560  (3.64×10^-106^) | 545  (6.07×10^-31^) |
| peptide receptor activity (MF) (114) | 113  (1.50×10^-6^) | 29  (1.23×10^-6^) |
| olfactory receptor activity (MF) (422) | 422  (8.21×10^-169^) | 6  (1.48×10^-119^) |

The values in the brackets are the P values of rank sum test between the classes in the first column and the intrinsic membrane, intrinsic to plasma membrane classes (excluding the common proteins). The numbers at the end of each class are the protein numbers of corresponding class.

**Fig. P The sulfur amino acid composition distribution of the total (blue), intrinsic membrane, intrinsic plasma membranes (purple) and other classes (dark red). The probability density curves were obtained by Gaussian-kernel smoothing of the individual data points.**

**Table D P Protein overlaps between the intrinsic membrane proteins and the classes with higher hydroxy amino acid composition**

| intrinsic to membrane (CC) | intrinsic to membrane (CC)(5385) | intrinsic to plasma membrane (CC) (1040) |
| --- | --- | --- |
| cell-cell adhesion (BP) 292 | 222  (0.39×10^-2^) | 66  (0.09) |
| neurological system process (BP) 947 | 657  (1.85×10^-17^) | 96  (8.48×10^-5^) |
| homophilic cell adhesion (BP) 139 | 138  (4.08×10^-6^) | 35  (1.72×10^-4^) |
| peptide receptor activity (MF)  114 | 113  (0.70×10^-2^) | 79  (0.04) |
| cytokine binding (MF)  113 | 97  (4.99×10^-5^) | 57  (7.15×10^-4^) |
| receptor activity (MF)  1715 | 1560  (7.89×10^-61^) | 545  (1.85×10^-16^) |
| olfactory receptor activity (MF) 422 | 422  (2.98×10^-69^) | 6  (6.73×10^-43^) |

**neurological system process**

The values in the brackets are the P values of rank sum test between the classes in the first column and the intrinsic membrane, intrinsic to plasma membrane proteins classes (excluding the common proteins). The numbers at the end of each class are the protein numbers of corresponding class.

**Fig Q The hydroxy amino acid composition distribution of the total (blue), intrinsic membrane, intrinsic plasma membranes (purple) and other classes (dark red). The probability density curves were obtained by Gaussian-kernel smoothing of the individual data points.**

**Table E Protein overlaps between the intrinsic membrane proteins and the classes with higher hydrophobicity**

|  | intrinsic to membrane (CC)  (5385) | intrinsic to plasma membrane (CC)  (1040) |
| --- | --- | --- |
| peptide receptor activity (MF)(114) | 113  (2.64×10^-21^) | 78  (2.83×10^-27^) |
| olfactory receptor activity (MF)(422) | 422  (5.87×10^-225^) | 6  (5.24×10^-176^) |
| metal ion transmembrane transporter activity (MF)(128) | 127  (6.13×10^-12^) | 60  (8.13×10^-16^) |
| amine transport (BP)(114) | 98  (1.96×10^-10^) | 47  (1.86×10^-13^) |
| organic acid transport (BP)(143) | 124  (1.04×10^-11^) | 57  (1.59×10^-15^) |
| neurological system process (BP)(947) | 657  (8.90×10^-39^) | 96  (3.53×10^-22^) |

The values in the brackets are the P values of rank sum test between the classes in the first column and the intrinsic membrane, intrinsic to plasma membrane proteins classes (excluding the common proteins). The numbers at the end of each class are the protein numbers of corresponding class.

**Fig. R The hydrophobicity distribution of the total (blue), intrinsic membrane, intrinsic plasma membranes (purple) and other classes (dark red). The probability density curves were obtained by Gaussian-kernel smoothing of the individual data points.**

**Table F Protein overlaps between the intrinsic membrane proteins and the classes with the higher *p*I**

|  | intrinsic membrane (CC)  (5385) |
| --- | --- |
| ribosome (CC) 196 | 3  (8.00×10^-66^) |
| mitochondrion (CC) 1254 | 297  (3.62×10^-26^) |
| mitochondrial matrix (CC) 150 | 1  (0.08) |
| mitochondrial membrane part (CC) 134 | 68  (7.98×10^-11^) |
| homophilic cell adhesion (BP) 139 | 138  (2.51×10^-60^) |
| microtubule-based movement (BP) 112 | 5  (1.40×10^-16^) |
| Golgi vesicle transport (BP) (131) | 36  (1.49×10^-17^) |
| MHC protein complex (CC) (118) | 114  (2.42×10^-17^) |

The values in the brackets are the P values of rank sum test between the classes in the first column and the intrinsic membrane proteins class (excluding the common proteins). The numbers at the end of each class are the protein numbers of corresponding class. The former four were enriched in the *p*I>9 group, while the later four classes were enriched in the *p*I<6 group.

..

**Fig. S The *p*I distribution of the total (blue), intrinsic membrane, (purple) and other classes (dark red, green). The probability density curves were obtained by Gaussian-kernel smoothing of the individual data points.**
