# Supplementary material for: The physical characteristics of human proteins in different biological functions
Source: PLoS One. 2017 May 1;12(5):e0176234. doi: 10.1371/journal.pone.0176234 (PMC5411090; doi:10.1371/journal.pone.0176234)
Supplement: S12 File — Table G-M. P-values of rank sum test between total and other classes with the higher hydrophobicity, pI, amino acid composition. (DOCX) [file pone.0176234.s012.docx]

**Table G *P*-values of rank sum test between total and other classes with the higher hydrophobicity**

| **intrinsic to membrane (CC)** | **5385** | **1040**  **(0)** | **113**  **(0)** | **422**  **(0)** | **127**  **(0)** | **98**  **(0)** | **124**  **(0)** | **657**  **(0)** |
| --- | --- | --- | --- | --- | --- | --- | --- | --- |
| **intrinsic to plasma membrane(CC)** | **1040** | **1,040** | **78**  **(6.60×10^-119^)** | **6**  **(1.17×10^-144^** | **60**  **(3.77×10^-127^)** | **47**  **(1.16×10^-131^)** | **57**  **(8.15×10^-128^)** | **96**  **(3.29×10^-125^)** |
| **peptide receptor activity (MF)** | **113** | **78**  **(1.86×10^-14^)** | **114** | **0**  **(5.54×10^-53^)** | **0**  **(5.54×10^-53^)** | **1**  **(1.67×10^-52^)** | **4**  **(4.02×10^-51^)** | **17**  **(1.52×10^-44^)** |
| **olfactory receptor activity (MF)** | **422** | **6**  **(1.09×10^-248^)** | **0**  **(3.88×10^-252^)** | **422** | **0**  **(3.88×10^-252^)** | **0**  **(3.88×10^-252^)** | **0**  **(3.88×10^-252^)** | **417**  **(0.13×10^-3^)** |
| **metal ion transmembrane transporter activity (MF)** | **127** | **60**  **(1.81×10^-21^)** | **0**  **(2.61×10^-47^)** | **0**  **(2.61×10^-47^)** | **128** | **22**  **(2.19×10^-36^)** | **27**  **(1.03×10^-33^)** | **7**  **(1.95×10^-40^)** |
| **amine transport (BP)** | **98**  **(0.48)** | **47**  **(3.40×10^-17^)** | **1**  **(3.61×10^-36^)** | **0**  **(1.25×10^-36^)** | **22**  **(5.08×10^-26^)** | **114** | **89**  **(6.89×10^-6^)** | **16**  **(5.28×10^-33^)** |
| **organic acid transport (BP)** | **124**  **(0.88)** | **57**  **(1.29×10^-22^)** | **4**  **(2.45×10^-45^)** | **0**  **(3.88×10^-47^)** | **27**  **(7.40×10^-34^)** | **89**  **(2.84×10^-16^)** | **143** | **15**  **(2.93×10^-43^)** |
| **neurological system process (BP)** | **657**  **(3.05×10^-5^)** | **96**  **(1.29×10^-142^)** | **17**  **(7.85×10^-159^)** | **417**  **(6.08×10^-13^)** | **7**  **(4.04×10^-163^)** | **16**  **(4.02×10^-162^)** | **15**  **(5.77×10^-162^)** | **947** |
|  | **intrinsic to membrane (CC)** | **intrinsic to plasma membrane (CC)** | **peptide receptor activity (MF)** | **olfactory receptor activity (MF)** | **metal ion transmembrane transporter activity (MF)** | **amine transport (BP)** | **organic acid transport (BP)** | **neurological system process (BP)** |

***** **The values in the brackets are the P values of rank sum test between the total and the classes in the first column (excluding the common proteins with the class in the bottom row). The numbers in the table are the overlap protein number of the two classes in the corresponding column and row.**

**Table H *P*-values of rank sum test between total and other classes with the higher *p*I**

| **intrinsic membrane (CC)** | **5,385** | **3**  **(6.92×10^-10^)** | **297**  **(3.84×10^-5^)** | **1**  **(5.88×10^-10^)** | **68**  **(1.09×10^-8^)** |
| --- | --- | --- | --- | --- | --- |
| **ribosome (CC)** | **3**  **(3.15×10^-66^)** | **196** | **81**  **(5.87×10^-39^)** | **0**  **(1.36×10^-65^)** | **1**  **(2.44×10^-65^)** |
| **mitochondrion (CC)** | **297**  **(1.08×10^-19^)** | **81**  **(1.28×10^-25^)** | **1254** | **147**  **(5.00×10^-36^)** | **131**  **(9.70×10^-30^)** |
| **mitochondrial matrix (CC)** | **1**  **(0.11×10^-2^)** | **0**  **(0.18×10^-2^)** | **147**  **(0.20)** | **150** | **11**  **(0.23×10^-2^)** |
| **mitochondrial membrane part (CC)** | **68**  **(2.23×10^-6^)** | **1**  **(9.48×10^-12^)** | **131**  **(0.53)** | **11**  **(2.84×10^-12^)** | **134** |
|  | **intrinsic membrane (CC)** | **ribosome (CC)** | **mitochondrion (CC)** | **mitochondrial matrix (CC)** | **mitochondrial membrane part (CC)** |

***** **The values in the brackets are the P values of rank sum test between the total and the classes in the first column (excluding the common proteins with the class in the bottom row). The numbers in the table are the overlap protein number of the two classes in the corresponding column and row.**

**Table I *P*-values of rank sum test between total and other classes with the lower *p*I**

| **intrinsic membrane (CC)** | **5,385** | **138**  **(1.86×10^-17^)** | **5**  **(4.65×10^-10^)** | **36**  **(3.06×10^-10^)** | **114**  **(1.42×10^-12^)** |
| --- | --- | --- | --- | --- | --- |
| **homophilic cell adhesion (BP)** | **138**  **(0.87)** | **139** | **0**  **(4.40×10^-53^)** | **0**  **(4.40×10^-53^)** | **0**  **(4.40×10^-53^)** |
| **microtubule-based movement (BP)** | **5**  **(1.35×10^-11^)** | **0**  **(2.19×10^-12^)** | **112** | **2**  **(3.09×10^-12^)** | **0**  **(2.19×10^-12^)** |
| **Golgi vesicle transport (BP)** | **36**  **(7.86×10^-14^)** | **0**  **(1.79×10^-12^)** | **2**  **(2.52×10^-12^)** | **131** | **0**  **(1.79×10^-12^)** |
| **MHC protein complex (CC)** | **114**  **(0.08)** | **0**  **(7.82×10^-11^)** | **0**  **(7.82×10^-11^)** | **0**  **(7.82×10^-11^)** | **118** |
|  | **intrinsic membrane (CC)** | **homophilic cell adhesion (BP)** | **microtubule-based movement (BP)** | **Golgi vesicle transport (BP)** | **MHC protein complex (CC)** |

***** **The values in the brackets are the P values of rank sum test between the total and the classes in the first column (excluding the common proteins with the class in the bottom row). The numbers in the table are the overlap protein number of the two classes in the corresponding column and row.**

**Table J *P*-values of rank sum test between total and other classes with the higher small amino acid composition**

| **intrinsic membrane (CC)** | **5385** | **1040**  **(0.56)** | **132**  **(0.05)** | **3**  **(0.002)** | **222**  **(0.66)** | **138**  **(0.22)** |
| --- | --- | --- | --- | --- | --- | --- |
| **intrinsic to plasma membrane (CC)** | **1040** | **1040** | **42**  **(1.84×10^-17^)** | **2**  **(1.93×10^-19^)** | **66**  **(1,20×10^-13^)** | **35**  **(2.93×10^-15^)** |
| **anchored to membrane (CC)** | **132** | **42**  **(1.17×10^-14^)** | **132** | **0**  **(1.80×10^-17^)** | **9**  **(4.52×10^-16^)** | **1**  **(3.86×10^-17^)** |
| **intermediate filament (BP)** | **3**  **(1.59×10^-26^)** | **2**  **(1.59×10^-27^)** | **0**  **(6.25×10^-28^)** | **184** | **0**  **(6.25×10^-^)** | **0**  **(6.25×10^-^)** |
| **cell-cell adhesion (BP)** | **222**  **(0.08)** | **66**  **(1.96×10^-30^)** | **9**  **(1.80×10^-41^)** | **0**  **(7.38×10^-43^)** | **292** | **139**  **(9.27×10^-11^)** |
| **homophilic cell adhesion (BP)** | **138** | **35**  **(4.61×10^-28^)** | **1**  **(3.52×10^-39^)** | **0**  **(1.39×10^-39^)** | **139** | **139** |
|  | **intrinsic membrane (CC)** | **intrinsic to plasma membrane (CC)** | **anchored to membrane (BP)** | **intermediate filament (BP)** | **cell-cell adhesion (BP)** | **homophilic cell adhesion (BP)** |

***** **The values in the brackets are the P values of rank sum test between the total and the classes in the first column (excluding the common proteins with the class in the bottom row). The numbers in the table are the overlap protein number of the two classes in the corresponding column and row.**

**Table K *P*-values of rank sum test between total and other classes with the higher aromatic amino acid composition**

| **intrinsic to membrane (CC)** | **5385** | **1040**  **(3.85×10^-222^)** | **179**  **(1.41×10^-232^)** | **657**  **(2.28×10^-158^)** | **100**  **(3.49×10^-236^)** | **113**  **(2.61×10^-236^)** | **113**  **(7.87×10^-238^)** | **192**  **(7.61×10^-222^)** | **422**  **(1.36×10^-175^)** |
| --- | --- | --- | --- | --- | --- | --- | --- | --- | --- |
| **intrinsic to plasma membrane (CC)** | **1040** | **1,040** | **17**  **(3.86×10^-46^)** | **96**  **(5.68×10^-41^)** | **6**  **(2.95×10^-48^)** | **3**  **(1.92×10^-48^)** | **70**  **(4.90×10^-38^)** | **8**  **(1.90×10^-47^)** | **6**  **(5.52×10^-48^)** |
| **lipid biosynthetic process (BP)** | **179**  **(1.38×10^-2^)** | **17**  **(1.34×10^-42^)** | **329** | **11**  **(2.44×10^-45^)** | **15**  **(8.42×10^-43^)** | **11**  **(2.09×10^-44^)** | **1**  **(1.34×10^-46^)** | **27**  **(9.16×10^-40^)** | **0**  **(5.07×10^-47^)** |
| **neurological system process (BP)** | **657**  **(0.13)** | **96**  **(1.61×10^-108^)** | **11**  **(9.45×10^-116^)** | **947** | **2**  **(7.56×10^-117^)** | **2**  **(9.42×10^-117^)** | **17**  **(5.47×10^-114^)** | **2**  **(9.42×10^-117^)** | **417**  **(3.34×10^-11^)** |
| **glycosylation**  **(BP)** | **100**  **(0.99)** | **6**  **(1.09×10^-27^)** | **15**  **(3.04×10^-24^)** | **2**  **(1.81×10^-28^)** | **114** | **36**  **(1.03×10^-19^)** | **0**  **(4.78×10^-29^)** | **95**  **(0.55)** | **0**  **(4.78×10^-29^)** |
| **UDP-glycosyltransferase activity (MF)** | **113**  **(0.002)** | **3**  **(2.07×10^-31^)** | **11**  **(7.51×10^-30^)** | **2**  **(5.24×10^-32^)** | **36**  **(2.12×10^-23^)** | **126** | **9**  **(1.00×10^-32^)** | **125**  **(0)** | **0**  **(1.00×10^-32^)** |
| **peptide receptor activity(MF)** | **113**  **(0.21)** | **70**  **(3.87×10^-9^)** | **1**  **(1.31×10^-25^)** | **17**  **(2.81×10^-22^)** | **0**  **(4.24×10^-26^)** | **0**  **(4.24×10^-26^)** | **114** | **0**  **(4.24×10^-26^)** | **0**  **(4.24×10^-26^)** |
| **transferase activity, transferring glycosyl groups (MF)** | **192**  **(4.81×10^-4^)** | **8**  **(1.92×10^-54^)** | **27**  **(2.33×10^-49^)** | **2**  **(1.18×10^-56^)** | **95**  **(2.69×10^-28^)** | **125**  **(5.34×10^-27^)** | **0**  **(2.39×10^-57^)** | **261** | **0**  **(2.39×10^-57^)** |
| **olfactory receptor activity (MF)** | **422** | **6**  **(1.31×10^-168^)** | **0**  **(1.43×10^-170^)** | **417**  **(8.32×10^-4^)** | **0**  **(1.43×10^-170^)** | **0**  **(1.43×10^-170^)** | **0**  **(1.43×10^-170^)** | **0**  **(1.43×10^-170^)** | **422** |
|  | **intrinsic to membrane (CC)** | **intrinsic to plasma membrane (CC)** | **lipid biosynthetic process (BP)** | **neurological system process (BP)** | **Glycosylation (BP)** | **UDP-glycosyltransferase activity (MF)** | **peptide receptor activity (MF)** | **transferase activity, transferring glycosyl groups (MF)** | **olfactory receptor activity (MF)** |

***** **The values in the brackets are the P values of rank sum test between the total and the classes in the first column (excluding the common proteins with the class in the bottom row). The numbers in the table are the overlap protein number of the two classes in the corresponding column and row.**

**Table L *P-*values of rank sum test between total and other classes with the higher sulfur amino acid composition**

| **intrinsic to membrane (CC)** | **5385** | **1040**  **(4.07×10^-30^)** | **27**  **(4.45×10^-40^)** | **64**  **(9.29×10^-39^)** | **44**  **(1.1.×10^-40^)** | **29**  **(1.01×10^-39^)** | **1560**  **(0.18)** | **113**  **(8.38×10^-36^)** | **422**  **(6.16×10^-11^)** |
| --- | --- | --- | --- | --- | --- | --- | --- | --- | --- |
| **intrinsic to plasma membrane (CC)** | **1040** | **1,040** | **20**  **(1.91×10^-15^)** | **33**  **(3.52×10^-14^)** | **16**  **(1.41×10^-15^)** | **14**  **(4.57×10^-15^)** | **545**  **(0.27)** | **29**  **(1.59×10^-10^)** | **6**  **(8.98×10^-15^)** |
| **response to bacterium (BP)** | **27**  **(9.23×10^-15^)** | **20**  **(2.73×10^-13^)** | **134** | **17**  **(6.94×10^-12^)** | **0**  **(1.18×10^-12^)** | **4**  **(7.22×10^-12^)** | **23**  **(2.06×10^-13^)** | **2**  **(1.26×10^-12^)** | **0**  **(1.18×10^-12^)** |
| **chemotaxis (BP)** | **64**  **(7.82×1015^-^)** | **33**  **(7.76×10^-14^)** | **17**  **(3.58×10^-15^)** | **173** | **0**  **(6.19×10^-16^)** | **57**  **(3.12×10^-^)** | **51**  **(7.23×10^-14^)** | **28**  **(9.57×10^-13^)** | **1**  **(1.34×10^-15^)** |
| **neurological system process (BP)** | **44**  **(0.12)** | **16**  **(0.04)** | **0**  **(0.11)** | **0**  **(0.11)** | **147** | **1**  **(0.14)** | **12**  **(0.13)** | **0**  **(0.11)** | **0**  **(0.11)** |
| **G-protein-coupled receptor binding (MF)** | **29**  **(1.12×10^-11^)** | **14**  **(1.54×10^-10^)** | **4**  **(1.45×10^-10^)** | **57**  **(0.01)** | **1**  **(1.30×10^-11^)** | **141** | **13**  **(1.37×10^-10^)** | **4**  **(1.55×10^-10^)** | **0**  **(2.60×10^-11^)** |
| **receptor activity (MF)** | **1560**  **(2.09×10^-7^)** | **545**  **(1.58×10^-122^)** | **23**  **(4.22×10^-136^)** | **51**  **(1.03×10^-133^)** | **12**  **(8.46×10^-137^)** | **13**  **(7.67×10^-135^)** | **1,715** | **114**  **(1.17×10^-126^)** | **422**  **(9.02×10^-40^)** |
| **peptide receptor activity (MF)** | **113**  **(0.12)** | **29**  **(0.05)** | **2**  **(9.21×10^-12^)** | **28**  **(1.58×10^-8^)** | **0**  **(8.74×10^-12^)** | **4**  **(6.05×10^-11^)** | **114** | **114** | **0**  **(8.75×10^-12^)** |
| **olfactory receptor activity (MF)** | **422** | **6**  **(2.56×10^-168^)** | **0**  **(5.02×10^-171^)** | **1**  **(1.58×10^-170^)** | **0**  **(5.02×10^-171^)** | **0**  **(5.02×10^-171^)** | **422** | **0**  **(5.02×10^-171^)** | **422** |
|  | **intrinsic to membrane (CC)** | **intrinsic to plasma membrane**  **(CC)** | **response to bacterium (BP)** | **chemotaxis (BP)** | **neurological system process (BP)** | **G-protein-coupled receptor binding (MF)** | **receptor activity (MF)** | **peptide receptor activity (MF)** | **olfactory receptor activity (MF)** |

***** **The values in the brackets are the P values of rank sum test between the total and the classes in the first column (excluding the common proteins with the class in the bottom row). The numbers in the table are the overlap protein number of the two classes in the corresponding column and row.**

**Table M *P-*values of rank sum test between total and other classes with the higher sulfur amino acid composition**

| **intrinsic to membrane (CC)**  **neurological system process** | **5385** | **1040**  **(1.61×10^-51^)** | **222**  **(2.77×10^-60^)** | **657**  **(1.02×10^-38^)** | **138**  **(2.37×10^-63^)** | **113**  **(4.95×10^-66^)** | **97**  **(6.96×10^-65^)** | **1560**  **(8.65×10^-14^)** | **422**  **(4.39×10^-40^)** |
| --- | --- | --- | --- | --- | --- | --- | --- | --- | --- |
| **intrinsic to plasma membrane (CC)** | **1040** | **1,040** | **66**  **(1.12×10^-23^)** | **96**  **(4.03×10^-25^)** | **35**  **(1.31×10^-25^)** | **79**  **(2.30×10^-23^)** | **57**  **(1.33×10^-24^)** | **545**  **(4.60×10^-6^)** | **6**  **(4.05×10^-27^)** |
| **cell-cell adhesion (BP)** | **222**  **(0.04)** | **66**  **(5.59×10^-8^)** | **292** | **16**  **(1.19×10^-13^)** | **139**  **(0.01)** | **0**  **(8.50×10^-13^)** | **0**  **(8.50×10^-13^)** | **44**  **(8.47×10^-11^)** | **0**  **(8.50×10^-13^)** |
| **neurological system process (BP)** | **657**  **(0.21)** | **96**  **(1.29×10^-42^)** | **16**  **(4.99×10^-46^)** | **947** | **3**  **(6.86×10^-45^)** | **17**  **(5.24×10^-45^)** | **1**  **(8.45×10^-45^)** | **542**  **(0.35)** | **417**  **(0.18)** |
| **homophilic cell adhesion (BP)** | **138**  **(0.29)** | **35**  **(7.34×10^-12^)** | **139** | **3**  **(1.27×10^-14^)** | **139** | **0**  **(8.57×10^-15^)** | **0**  **(8.57×10^-15^)** | **14**  **(5.28×10^-13^)** | **0**  **(8.57×10^-15^)** |
| **peptide receptor activity (MF)** | **113**  **(0.30)** | **79**  **(0.03)** | **0**  **(5.01×10^-8^)** | **17**  **(4.70×10^-8^)** | **0**  **(5.01×10^-8^)** | **114** | **24**  **(6.03×10^-6^)** | **114**  **0** | **0**  **(5.01×10^-8^)** |
| **cytokine binding (MF)** | **97**  **(0.97)** | **57**  **(9.05×10^-7^)** | **0**  **(6.47×10^-11^)** | **1**  **(1.48×10^-10^)** | **0**  **(6.74×10^-11^)** | **24**  **(9.70×10^-9^)** | **113** | **100**  **(0.63)** | **0**  **(6.74×10^-11^)** |
| **receptor activity (MF)** | **1560**  **(0.54)** | **545**  **(3.05×10^-89^)** | **44**  **(7.52×10^-109^)** | **542**  **(8.44×10^-46^)** | **14**  **(6.33×10^-109^)** | **114**  **(5.17×10^-105^)** | **100**  **(3.75×10^-101^)** | **1715** | **422**  **(4.31×10^-49^)** |
| **olfactory receptor activity (MF)** | **422** | **6**  **(2.00×10^-88^)** | **0**  **(1.18×10^-89^)** | **417**  **（0.07）** | **0**  **(1.18×10^-89^)** | **0**  **(1.18×10^-89^)** | **0**  **(1.18×10^-89^)** | **422** | **422** |
|  | **intrinsic to membrane (CC)** | **intrinsic to plasma membrane (CC)** | **cell-cell adhesion (BP)** | **neurological system process (BP)** | **homophilic cell adhesion (BP)** | **peptide receptor activity (MF)** | **cytokine binding (MF)** | **receptor activity (MF)** | **olfactory receptor activity (MF)** |

***** **The values in the brackets are the P values of rank sum test between the total and the classes in the first column (excluding the common proteins with the class in the bottom row). The numbers in the table are the overlap protein number of the two classes in the corresponding column and row.**
